# On a mission to block transmission

**DOI:** 10.7554/eLife.35246

**Published:** 2018-02-27

**Authors:** Amanda Ross, Nicolas MB Brancucci

**Affiliations:** 1 Department of Epidemiology and Public HealthSwiss Tropical and Public Health InstituteBaselSwitzerland; 2 University of BaselBaselSwitzerland; 3 Department of Molecular Parasitology and Infection BiologySwiss Tropical and Public Health InstituteBaselSwitzerland

**Keywords:** malaria, gametocytes, Plasmodium falciparum, transmission, *P. falciparum*

## Abstract

The controlled infection of volunteers with *Plasmodium falciparum* parasites could provide a platform to evaluate new drugs and vaccines aimed at blocking malaria transmission.

**Related research article** Reuling IJ, van de Schans LA, Coffeng LE, Lanke K, Meerstein-Kessel L, Graumans W, van Gemert G, Teelen K, Siebelink-Stoter R, van de Vegte-Bolmer M, de Mast Q, van der Ven AJ, Ivinson K, Hermsen CC, de Vlas S, Bradley J, Collins KA, Ockenhouse CF, McCarthy J, Sauerwein RW, Bousema T. 2018. A randomized feasibility trial comparing four antimalarial drug regimens to induce *Plasmodium falciparum* gametocytemia in the controlled human malaria infection model. *eLife* **7**:e31549. doi: 10.7554/eLife.31549

Life inside a human host is fraught with danger. *Plasmodium falciparum* malaria parasites need to be highly adapted to compete for resources and to take their chances with the immune system. The same degree of specialization is needed to survive inside the mosquito, and for the transitions from human to mosquito and vice versa. These seemingly incompatible needs are met by a life cycle that involves morphing into a series of different forms.

Under the microscope, a blood sample may reveal different stages of the parasite: asexual parasites that replicate and prolong the infection, and sexual stages called gametocytes, the form that transmits the infection from human to mosquito. Both male and female gametocytes arise when a small proportion of asexual parasites stop replication and commit to sexual development instead. After maturation they are either taken up by a feeding mosquito or die. Sex and transmission are inextricably linked. Fertilization occurs inside the mosquito gut within an hour of feeding and leads to several developmental phases inside the insect vector, after which parasites are ready to infect a new human host (Sinden, 2017).

Most licensed antimalarial drugs target the asexual forms of the parasite. These stages are the most abundant and cause the symptoms of malaria (Sinden, 2017; Nilsson et al., 2015). As the burden of disease has declined in many regions and the focus is shifting towards malaria elimination, the searchlight beam now falls onto other stages. On paper, gametocytes are a good target to break the circle of transmission: they are the only form capable of infecting mosquitoes and their low number creates a population bottleneck.

However, efforts to introduce new drugs and vaccines that target gametocytes and prevent malaria transmission face an obstacle: there is no method to directly evaluate their success in humans. Clinical studies in malaria patients can demonstrate the effect of drugs on asexual parasites, but often fail to capture the dynamics of gametocytes for simple reasons. Gametocytes generally occur at very low densities and are often missed by standard detection methods. They sequester away from the blood stream for eight to twelve days as they mature, and often are not seen at the time of illness. Consequently, the evaluation of drugs is mostly limited to unnatural conditions, such as measuring gametocyte densities in cell cultures or artificially feeding blood infected with gametocytes to mosquitoes.

Now, in eLife, Robert Sauerwein, Teun Bousema and colleagues – including Isaie Reuling as first author – report a promising method to fill this void (Reuling et al., 2018). The researchers – who are based at the Radboud University Medical Center and other universities and non-profit organizations in the UK, United States, Australia and the Netherlands – drew on recent scientific developments in sensitive molecular diagnostic methods, the stage-specificity of drugs and the resurgence of interest in human challenge studies (Farid et al., 2017; Stanisic et al., 2018; Stone et al., 2017).

Reuling et al. deliberately infected volunteers with *P. falciparum* using *Anopheles* mosquitoes. The ensuing asexual parasite densities were controlled and eventually cleared using different drugs while allowing the gametocytes to continue development under safe conditions. All of the volunteers yielded circulating gametocytes.

A crucial question is how well this method of generating gametocytes in humans would work to evaluate a compound that blocks transmission. Effects on gametocyte densities can be shown (Collins et al., 2018). However, such densities are only loosely related to transmission success (Churcher et al., 2013). The most relevant measure would be whether or not feeding mosquitoes become infected. Reuling et al. observed that the low gametocyte numbers in the volunteers only sporadically infected mosquitoes, and are aware that this renders conclusions about transmission-blocking activity impossible.

Changing certain elements of the study setup, for example using different drugs, treatment doses, *P. falciparum* laboratory strains or mosquito species, may overcome this issue. Another possibility could be to enhance gametocyte production by inducing sexual commitment. However, we are only beginning to understand the triggers for this process (Bechtsi and Waters, 2017). Recently, a human lipid was shown to inhibit sexual commitment (Brancucci et al., 2017). Interfering with the underlying regulatory pathway could be exploited in the future. But regardless of how these problems are tackled, questions of how well the system translates into real life settings would need to be addressed carefully.

A side-effect of piloting the method of controlled human infection is the insight into the secret lives of gametocytes. Previously, the most detailed studies of gametocyte dynamics had been made when deliberate infections were used to treat neurosyphilis patients at a time before antibiotics were available (Snounou and Pérignon, 2013). By using highly sensitive and sex-specific detection methods, Reuling et al. add to the pool of knowledge and provide evidence that mature male and female gametocytes first appear in the blood at different times, and that female gametocytes circulate in the blood for longer. The early appearance of mature gametocytes in the peripheral blood of the volunteers further suggests that a fraction of the first wave of parasites entering the blood stream can already be sexually committed.

A system to evaluate potential transmission-blocking strategies *in vivo* is hugely appealing. In future, an optimized setup that can achieve a higher proportion of infected mosquitoes may help to discover new ways to prevent malaria transmission. The work of Reuling et al. has opened a door.

**Figure 1.**
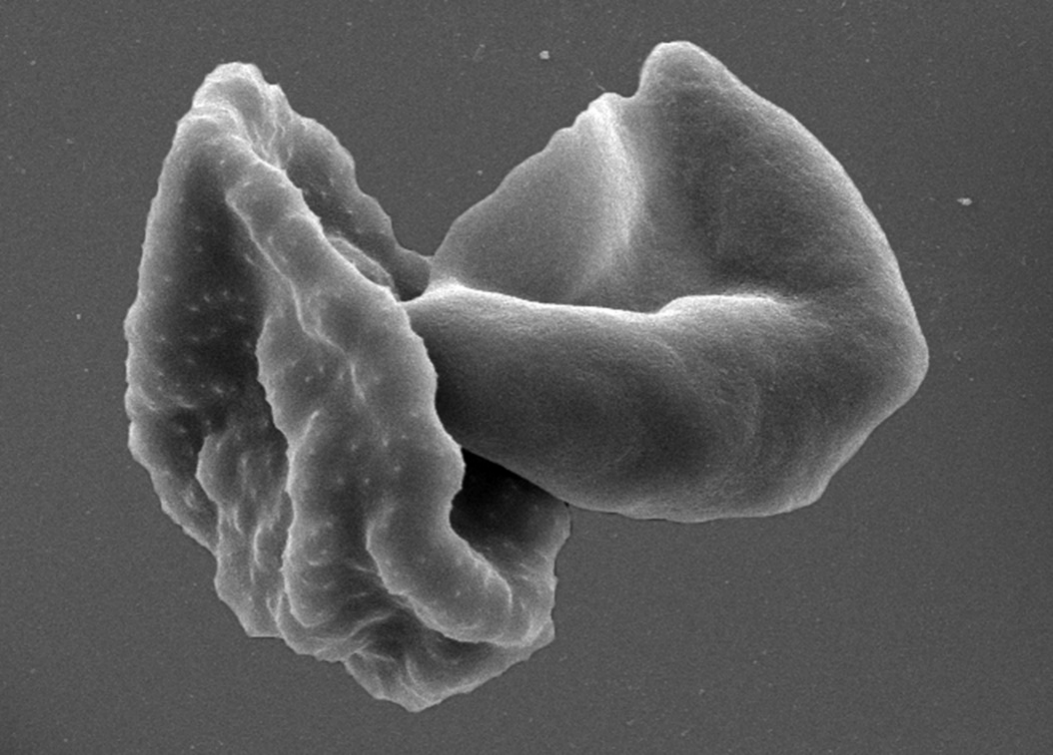
Red blood cells infected with *Plasmodium falciparum* parasites. Malaria parasites have a complex life cycle and different forms of the parasites can be found in the blood. This scanning electron micrograph shows red blood cells infected with the parasite: an asexual stage on the left and a mature gametocyte on the right. Only the gametocytes can infect mosquitoes.
